# Gemcitabine, cisplatin and methylprednisolone (GEM-P) is an effective salvage regimen in patients with relapsed and refractory lymphoma

**DOI:** 10.1038/sj.bjc.6602514

**Published:** 2005-04-05

**Authors:** M Ng, J Waters, D Cunningham, I Chau, A Horwich, M Hill, A R Norman, A Wotherspoon, D Catovsky

**Affiliations:** 1 Department of Medicine, Royal Marsden Hospital, London and Surrey, UK; 2 Department of Clinical Oncology, Royal Marsden Hospital, London and Surrey, UK; 3 Department of Medical Oncology, Kent Oncology Centre, Maidstone, Kent, UK; 4 Department of Computing, Royal Marsden Hospital, London and Surrey, UK; 5 Department of Histopathology, Royal Marsden Hospital, London and Surrey, UK; 6 Academic Department of Haematology, Royal Marsden Hospital, London and Surrey, UK

**Keywords:** gemcitabine, cisplatin, relapsed, refractory, lymphoma

## Abstract

There is currently no standard salvage chemotherapy regimen in relapsed and refractory lymphoma. Gemcitabine is a novel nucleoside analogue, which acts synergistically with cisplatin both *in vitro* and in clinical studies. We evaluated the combination of gemcitabine, cisplatin and methylprednisolone (GEM-P) in 41 heavily pretreated patients with relapsed and refractory Hodgkin's and non-Hodgkin's lymphoma. The best-achieved response rate (RR) was 79% (95% CI 64–91), with a complete RR of 21%. In patients with chemo-resistant disease, the RR was 63%. Myelosuppression was the main toxicity, the incidence of Grade 3 or 4 anaemia, neutropenia and thrombocytopenia was 17.1, 61.0 and 53.7% respectively. Only one patient had neutropenic sepsis and none of the patients suffered from haemorrhage. Grade 3 or 4 nonhaematological toxicity was minimal and stem cell mobilisation was not inhibited. GEM-P is an effective salvage regimen and its use prior to autologous stem cell transplant warrants further investigation.

Several advances in the last decade in the treatment of Hodgkin's and non-Hodgkin's lymphoma (NHL) have led to improved rates of cure. The combination of chemotherapy with radiotherapy for limited stage Hodgkin's lymphoma (HL) and the introduction of dose-intensified BEACOPP (bleomycin, etoposide, doxorubicin, cyclophosphamide, vincristine, procarbazine and prednisolone) for poor prognosis advanced stage HL have improved relapse-free survival in patients (Canellos *et al*, 1992; Noordijk *et al*, 1997; Sieber *et al*, 2002). The adoption of ABVD (doxorubicin, bleomycin, vinblastine and dacarbazine) as standard first-line therapy for advanced HL has led to higher response rates (RRs) with less treatment related toxicity (Canellos *et al*, 1992). The addition of rituximab to conventional chemotherapy has improved RRs and time to treatment failure in follicular lymphoma (Marcus *et al*, 2003), while also improving overall survival in diffuse large B-cell lymphoma (DLBCL) (Coiffier *et al*, 2002). Despite these improvements in treatment, approximately 50% of patients with advanced disease will relapse or fail treatment with first line chemotherapy (Fisher *et al*, 1994; Canellos and Niedzwiecki, 2002; Coiffier *et al*, 2002). However, salvage chemotherapy followed by high-dose chemotherapy and autologous stem cell transplant (ASCT) in patients with chemo-sensitive HL and aggressive NHL can lead to long-term remission in 46–55% of patients (Linch *et al*, 1993; Philip *et al*, 1995; Schmitz *et al*, 2002).

At present, there is no accepted standard salvage chemotherapy. Regimens that are commonly employed are usually cisplatin based such as DHAP (dexamethasone, cytosine arabinoside and cisplatin) (Velasquez *et al*, 1988) and ESHAP (etoposide, methylprednisolone, cytosine arabinoside and cisplatin) (Velasquez *et al*, 1994) or utilise an ifosfamide–etoposide backbone such as ICE (ifosfamide, carboplatin and etoposide) (Moskowitz *et al*, 1999) or MINE (mitoguanzone, ifosfamide, vinorelbine, etoposide) (Ferme *et al*, 1995). Gemcitabine is a novel nucleoside analogue, which is active in solid tumours such as pancreatic, ovarian and non-small cell lung cancer (Burris *et al*, 1997; Manegold *et al*, 1997). *In vitro* studies of gemcitabine have demonstrated its ability to circumvent multi-drug resistance (MDR) secondary to increased P-glycoprotein and MDR protein 1 overexpression (Bergman *et al*, 2003). These MDR cells are associated with increased deoxycytidine kinase activity and reduced deoxycytidine deaminase activity leading to accumulation and increase sensitivity to gemcitabine (Bergman *et al*, 2001). Preclinical studies of gemcitabine and cisplatin have demonstrated synergy both *in vitro* and *in vivo* (Peters *et al*, 1995). Phase I studies evaluating this combination in relapsed and refractory HL and NHL have also reported higher RRs (Aviles *et al*, 2004; Emmanouilides *et al*, 2004) compared to either agent given as monotherapy (Fossa *et al*, 1999; Santoro *et al*, 2000; Savage *et al*, 2000; Zinzani *et al*, 2000). The mechanisms underlying this synergy may be due to increased incorporation of gemcitabine into DNA and RNA, and increased cisplatin–DNA adduct formation by inhibition of exonuclease and DNA repair (van Moorsel *et al*, 1999). Given their synergism, absence of significant overlapping toxicities and non-cross-resistance with other regimens, gemcitabine in combination with cisplatin, presents an attractive treatment option. Furthermore, treatment with gemcitabine and cisplatin have been shown to induce responses in patients with ovarian carcinoma previously resistant to cisplatin or gemcitabine (Rose *et al*, 2003). We previously reported a RR of 80% with GEM-P in 20 patients with relapsed and refractory lymphoma, having met our target of excluding a lower RR of 20% (Chau *et al*, 2003). Following our initial report, we expanded our study to recruit a further 20 patients and now present the results for all 42 patients, having established a more precise estimate of the effectiveness of this regimen.

## MATERIALS AND METHODS

The study was approved by the institution research and ethics committees. Signed, written informed consent was obtained from each patient.

### Patients selection and evaluation

Patients aged over 18 years with histology-proven diagnosis of HD and NHL were eligible (previous diagnoses were reformulated according to the World Health Organization (WHO) classification of lymphoid neoplasms) (Harris *et al*, 2000). They had to have documented relapse or progressive disease following previous chemotherapy; uni-dimensional measurable disease on computed tomography (CT) scan; WHO performance status 0–2; no previous therapy with either a platinum compound or gemcitabine. Adequate bone marrow and hepatic function with a glomerular filtration rate of >60 ml min^−1^ was also required. Patients with acquired immunodeficiency syndrome (AIDS)-related lymphoma, positive human immunodeficiency virus serology and hearing impairment were excluded. Pretreatment staging was by CT scans of the chest, abdomen, pelvis (and neck if indicated); bone marrow biopsy for all NHL patients and for HD patients with documented bone marrow infiltration in the past or clinical suspicion of bone marrow involvement with current relapse.

### Chemotherapy regimen

Gemcitabine (1000 mg m^−2^) was delivered as an intravenous infusion over 30 min on days 1, 8 and 15. Cisplatin (100 mg m^−2^) was given over 4 h on day 15 only. Cisplatin was started 4 h after gemcitabine infusion. Pre- and post-chemotherapy hydration was given on the day of cisplatin administration. Patients also received methylprednisolone 1000 mg either orally or intravenously on days 1–5. The cycle was repeated every 28 days.

### Dose modifications

Toxicity was assessed according to WHO toxicity criteria. On the day of treatment, if the neutrophil count was 0.5–0.9 × 10^9^ l^−1^ and/or the platelet count was 50–74 × 10^9^ l^−1^, the gemcitabine dose was reduced by 25%. If the neutrophil count was <0.5 × 10^9^ l^−1^ and/or the platelet count was <50 × 10^9^ l^−1^, both gemcitabine and cisplatin were withheld. Patients with febrile neutropenia were given granulocyte-colony-stimulating factor (G-CSF) and both gemcitabine and cisplatin were withheld until haematological recovery, with subsequent dose reductions by 25%. In the event of grade 3 or 4 nonhaematological toxicity, gemcitabine and cisplatin doses were reduced by 25 and 50%, respectively. With the first occurrence of transient tinnitus, cisplatin dose was reduced to 80 mg m^−2^ and with a second occurrence, the dose was further reduced to 60 mg m^−2^. Cisplatin was discontinued in patients with unresolved or recurrent tinnitus. Patients who developed renal impairment had their cisplatin dose adjusted according to their EDTA clearance.

### Dose intensity

Owing to the heterogeneity of the study population, the planned treatment duration was different between patients. Dose intensity for each drug was calculated using the total administered dose density for all patients divided by the total planned dose density for all patients.

### Autologous stem cell transplantation

Patients undergoing high-dose chemotherapy and ASCT following GEM-P were required to have a stem cell harvest of >1.5 × 10^6^ CD34+ cells kg^−1^. The myeloablative regimens used were melphalan and etoposide (ME) or melphalan, carmustine and etoposide (MBE).

### Evaluation of response

Tumour response was measured by CT scan after every two cycles and at the end of treatment in accordance to the Response Evaluation Criteria in Solid Tumours (RECIST) Guidelines (Therasse *et al*, 2000). Bone marrow biopsy was repeated at the end of treatment for patients with lymphomatous involvement prior to treatment. Complete response (CR) was defined as the disappearance of all target lesions, without the appearance of new lesion(s) and absence of lymphomatous involvement on bone marrow biopsy. Partial response (PR) was defined as at least a 30% decrease in the sum of the longest diameter of target lesions, taking as reference the baseline sum of the longest diameters. Progressive disease (PD) was defined as at least a 20% increase in the sum of the longest diameter of target lesions, taking as reference the smallest sum of the longest diameters recorded since treatment started or by the appearance of new lesions. Stable disease (SD) was defined as neither sufficient shrinkage to qualify for PR nor sufficient increase to qualify for PD, taking as reference the smallest sum of the longest diameters since the treatment started. The RECIST criteria were chosen because of the heterogeneity of the patient population, which included both patients with HL and NHL, as well as those with visceral disease. In these patients, neither the Cotswold criteria (Lister *et al*, 1989) nor the International Workshop NHL response criteria (Cheson *et al*, 1999) could be strictly applied.

### Statistical analysis

The trial was initially performed using a Gehan's two-stage design for estimating the RR. However, after recruiting 21 patients, the trial was stopped as the target of excluding a lower of 20% was achieved. The trial was then expanded to recruit 42 patients in total to obtain a point estimate of the RR with a confidence interval of ±15%.

Progression-free survival (PFS) and overall survival (OS) were estimated using the Kaplan–Meier method. Progression-free survival was calculated from the start of chemotherapy to the date of relapse, progression or last follow-up. Overall survival was estimated from the start of chemotherapy to the date of death from any cause.

## RESULTS

Between January 2001 and July 2003, 42 patients were recruited from two centres into the study. One patient became ineligible during treatment, as the relapse was found to be from a new colorectal cancer, and has been excluded from analysis. One patient had a protocol violation as he received rituximab at cycle 4, following an earlier partial response but has been included in the analysis. The baseline characteristics of patients are shown in (Table 1). The median age was 42 years, with the majority of patients having nodular sclerosing HL (39%) or DLBCL (24%). Nearly three-quarters of patients had advanced stage disease, while 26 patients (63%) had an elevated lactate dehydrogenase (LDH) and 13 patients (32%) had B symptoms. Table 2 lists their disease status and the previous treatments they received at study entry. The median number of prior chemotherapy regimens was 2 with a maximum of 5. Six patients had previously undergone ASCT, seven patients had received rituximab while 18 (44%) had been treated with radiotherapy. In all, 24 patients (59%) were in second or subsequent relapse, 29 patients (71%) had primary progressive disease or relapsed within a year and 19 patients (46%) had chemo-resistant disease.

### Response to treatment and survival

**Figure 1:**
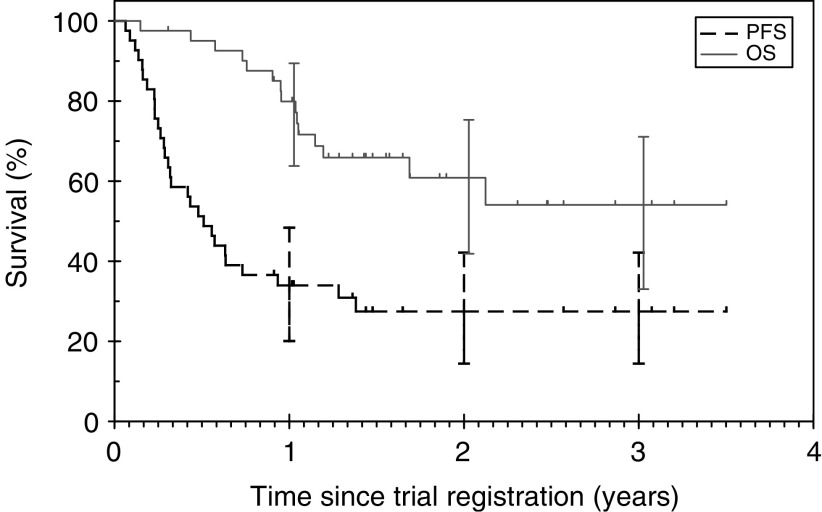
Overall survival and progression-free survival.

A total of 39 patients were evaluable for response, as two patients did not have measurable disease. The best-achieved RR was 79% (95% confidence interval (CI) 64–91) with eight patients (21%) obtaining a CR and 23 patients (58%) obtaining a PR. GEM-P was active across the spectrum of histological subtypes, and in chemo-resistant disease the RR was 63% (Table 3). In first relapse and primary progressive disease the RR was 78 and 50%, respectively. After a median follow-up of 571 days, 26 patients are still alive. The median PFS is 186 days (95% CI 117–255) and estimated overall survival at 3 years is 54% (95% CI 33–71) (Figure 1).

### Dose intensity and treatment toxicity

A total of 137 cycles were administered and the median number of cycles per patient was 3 (range 1–6). The reasons for cessation of treatment were a planned decision (*n*=20), treatment failure (*n*=16), patient choice (*n*=3) and toxicity (*n*=2). The three patients who chose to stop treatment were psychologically unable to cope with further chemotherapy. Two other patients stopped treatment because of lethargy and tinnitus. In all, 24 patients (59%) experienced dose delays primarily due to myelosuppression, while eight patients received G-CSF. The dose intensity for gemcitabine and cisplatin was 81.2 and 72.3%. respectively. Eight patients discontinued cisplatin, three from ototoxicity, two from lethargy, two from dehydration with renal impairment and one for an unspecified reason. Toxicity from treatment as assessed by WHO criteria is listed in (Table 4). The incidence of grade 3 or 4 neutropenia and thrombocytopenia was 61.0% and 53.7%, respectively; however, only one patient had neutropenic sepsis and none suffered from haemorrhage. Grade 3 or 4 nonhaematological toxicity was limited to nausea and vomiting, diarrhoea, neuropathy and lethargy. Their incidence was 2.4%, 4.8%, 2.4% and 2.5%, respectively. The patient with grade 3 neuropathy at the start of treatment had cord compression, which improved following a complete response to chemotherapy.

### Treatment consolidation

Twenty-two patients were planned for high-dose consolidation with ASCT following GEM-P. Of these patients, seven were in first relapse, six had primary refractory disease, seven were in second relapse and two were in third relapse. Eight patients underwent ASCT , the remaining patients did not undergo ASCT due to treatment failure (*n*=10), patient choice (*n*=2), cardiac insufficiency (*n*=1) and subclavian vein thrombosis (*n*=1). Stem cell harvesting was performed in nine patients, the median CD 34+ cell count was 2.25 × 10^6^ kg^−1^ (range 1.65–3.21). The median time to engraftment to achieve a neutrophil count >0.5 × 10^9^ l^−1^ was 11 days (10–18) and to achieve a platelet count >20 × 10^9^ l^−1^ was 10 days (8–12). There was no ASCT treatment-related mortality. Following ASCT, five patients remain in remission while three patients have relapsed of whom one has died. Autologous stem cell transplant was not planned in the other 19 patients because of previous ASCT (*n*=6), indolent histology (*n*=6), multiply relapsed disease (*n*=5), age (*n*=1) and physician's decision (*n*=1). Three of these patients received involved field radiotherapy as consolidation following treatment with GEM-P, one of whom has relapsed and all three are still surviving.

## DISCUSSION

The RRs that we have observed with GEM-P is encouraging and comparable to other salvage regimens (Velasquez *et al*, 1988, 1994; Moskowitz *et al*, 1999). Of particular importance, these responses are seen in a heavily pretreated population with several poor prognostic features such as chemo-resistant disease (Josting *et al*, 2000), relapse within a year (Brice *et al*, 1997; Guglielmi *et al*, 1998), advanced disease stage (Blay *et al*, 1998) and previous ASCT. Response to salvage chemotherapy is important prior to ASCT, as it is correlated with survival post-transplant (Philip *et al*, 1987; Brice *et al*, 1997). Another significant finding was that treatment with GEM-P did not appear to inhibit stem cell harvesting, although this was not an end point of the study. Myelosuppression was the main toxicity leading to treatment delays; however, only one patient developed neutropenic sepsis and eight patients received G-CSF. This may have been accentuated by half of the patients having receiving two or more lines of prior chemotherapy. From our ongoing experience with GEM-P, by administering G-CSF on the 5th and 6th day after each dose of gemcitabine, delays from neutropenia can be circumvented. Grade 3 or 4 nonhaematological toxicity was minimal and we did not observe any cases of pulmonary toxicity. Several studies have reported the development of severe pulmonary toxicity in association with gemcitabine. The incidence of pulmonary toxicity has been estimated to vary from 0 to 5% (Roychowdhury *et al*, 2002; Barlesi *et al*, 2003) and may be increased by administering bleomycin (Friedberg *et al*, 2003; Bredenfeld *et al*, 2004) or docetaxel (Dunsford *et al*, 1999; Kouroussis *et al*, 2004) in combination with gemcitabine. In the above-mentioned study by Bredenfeld *et al*, gemcitabine was given on days 1 and 4 of each 3 weekly cycle and the dose was increased from 800 to 1500 mg m^−2^. These differences may account for the absence of pulmonary toxicity seen in our study. All of the patients who developed renal impairment improved, with resolution in most. Only two patients required discontinuation of treatment with cisplatin, following which their renal impairment resolved to Grade 0 and 1, respectively. The most common nonhaematological toxicity was lethargy, which led to two patients stopping their treatment. As a result, patients who were suitable for ASCT received a median of 2 cycles of chemotherapy (range 2–4). In addition, the GEM-P regimen does not contain alkylating agents or etoposide, thereby reducing the risk of secondary haematological malignancies in patients undergoing potentially curative treatment.

Future directions with the GEM-P regimen would look to increase its efficacy and reduce the toxicity of treatment. Rituximab has been shown to enhance cytotoxicity with cisplatin and overcome resistance to cisplatin and gemcitabine *in-vitro* (Demidem *et al*, 1997; Emmanouilides *et al*, 2002). Combination of rituximab with GEM-P may lead to improved CR rates similar to that seen with rituximab and ICE (Kewalramani *et al*, 2004). This may be important as some studies suggest that patients who undergo ASCT in CR do better than those in PR (Moskowitz *et al*, 1999). Oxaliplatin, which is a diaminocyclohexane platinum, has an RR of 40% in patients with relapsed and refractory NHL, and is also active in patients resistant to cisplatin (Germann *et al*, 1999). As oxaliplatin is not associated with nephrotoxicity and ototoxicity (Chau *et al*, 2001), it may be an alternative for patients who are not suitable or who discontinue treatment with cisplatin because of nephrotoxicity and ototoxicity. Another way to enhance the cytotoxicity of gemcitabine is to administer it as a fixed dose rate infusion, this has been shown to improve drug delivery into tumour cells (Grunewald *et al*, 1992). Phase I studies of fixed dose rate gemcitabine infusion in combination with cisplatin and dexamethasone have reported an RR of 45% in relapsed and refractory lymphoma (Emmanouilides *et al*, 2004).

Gemcitabine in combination with cisplatin and methylprednisolone is an effective salvage regimen in patients with relapsed and refractory lymphoma. As it is well tolerated, with minimal severe nonhaematological toxicity and does not appear to inhibit stem cell harvesting, it should be considered for use as reinduction therapy prior to ASCT. Further studies to improve its efficacy and to compare it with existing salvage regimens are warranted.

## Figures and Tables

**Table 1:** Patient baseline characteristics

**Characteristics**	**Number of patients**
Eligible patients	41
*Age (years)*	
Median	42
Range	17–68

*Sex*	
M:F	29:12

*WHO performance status*	
0	13
1	23
2	5

*B symptoms*	
Yes	13
No	28

*Stage*	
1–2	11
3–4	30

*LDH*	
⩽ULN	15
>ULN	26

*Extra-nodal sites*	
<2	35
⩾2	6

Histology	
*Hodgkin's lymphoma*	
Nodular sclerosing	16
Lymphocyte predominant	1

*B-cell type*	
Follicular Lymphoma Grade 1–3A	3
Diffuse large B-cell lymphoma	10
T-cell-rich B-cell lymphoma	2
Mantle cell lymphoma	1

*T-cell type*	
T/NK cell	1
Angioimmunoblastic lymphoma	1
Anaplastic large cell lymphoma	3
Enteropathy associated lymphoma	1

**Table 2:** Disease status at trial entry and previous treatments

**Previous treatment details**	
Prior lines of chemotherapy	
*Median (range)*	2 (1–5)
1	18
2	12
3⩾	11

*Prior high-dose chemotherapy*	
Yes	6
No	35

*Prior radiotherapy*	
Yes	18
No	23

*Prior rituximab*	
Yes	7
No	34

*Disease status*	
Primary progressive^a^	8 patients
1st relapse	9 patients
2nd relapse	11 patients
3rd and subsequent relapse	13 patients

*Remission duration* b	
< 1 year	29 patients
> 1 year	12 patients
Chemosensitive^c^	22
Chemoresistant^d^	19

a Disease progression during or within 90 days of completion of induction treatment.

b Disease-free interval from end of last treatment till relapse.

c Response to prior chemotherapy lasting greater than 90 days from completion of treatment.

d Disease progression during or within 90 days of completion of last chemotherapy.

**Table 3:** Best-achieved response rates

	**No of patients**	**CR**	**PR**	**SD**	**PD**	**RR % (95% CI)**
Evaluable patients	39^a^	8	23	4	4	79 (64–91)
Primary progressive	8	0	4	2	2	50 (16–84)
1st relapse	8	2	5	1	0	88 (47–100)
2nd and subsequent relapse	23	6	14	1	2	87 (66–97)
Chemosensitive	20	5	14	1	0	95 (75–100)
Chemoresistant	19	3	9	3	4	63 (38–84)
Hodgkin's lymphoma	17	2	12	1	2	82 (57–96)
DLBCL	1	2	5	2	2	64 (31–89)
Follicular lymphoma	3	1	1	1	0	67 (9–99)
Mantle cell lymphoma	2	0	2	0	0	100 (16–100)
T-cell lymphoma	6	3	3	0	0	100 (29–100)

a Two patients were not included in response evaluation due to absence of measurable disease.

**Table 4:** Toxicity profile

	**Maximum WHO toxicity grade which occurred (% of patients)**			
	**0**	**1 or 2**	**3**	**4**
Anaemia	9.8	73.1	14.6	2.5
Leucopenia	4.9	36.6	31.7	26.8
Neutropenia	17	22	31.7	29.3
Thrombocytopenia	12.2	34.1	29.3	24.4
Infection	51.2	46.4	0	2.4
Haemorrhage	100	0	0	0
Nausea and vomiting	43.9	53.7	2.4	0
Stomatitis	70.7	29.3	0	0
Diarrhoea	63.4	31.8	2.4	2.4
Neuropathy	53.7	43.9	2.4	0
Ototoxicity	58.5	41.4	0	0
Renal impairment	56.1	43.9	0	0
Lethargy	32.5	65	2.5	0
Aloplecia	80.5	19.5	0	0

## References

[bib1] Aviles A, Neri N, Huerta-Guzman J, Fernandez R (2004) Gemcitabine and cisplatin in refractory malignant lymphoma. Oncology 66: 197–200 15218310 10.1159/000077995

[bib2] Barlesi F, Doddoli C, Gimenez C, Greillier L, Lima G, Kleisbauer JP (2003) Acute pulmonary toxicity due to gemcitabine: a role for asbestos exposure? Rev Mal Respir 20: 201–206 12844017

[bib3] Bergman AM, Munch-Petersen B, Jensen PB, Sehested M, Veerman G, Voorn DA, Smid K, Pinedo HM, Peters GJ (2001) Collateral sensitivity to gemcitabine (2′,2′-difluorodeoxycytidine) and cytosine arabinoside of daunorubicin- and VM-26-resistant variants of human small cell lung cancer cell lines. Biochem Pharmacol 61: 1401–1408 11331076 10.1016/s0006-2952(01)00627-x

[bib4] Bergman AM, Pinedo HM, Talianidis I, Veerman G, Loves WJ, van der Wilt CL, Peters GJ (2003) Increased sensitivity to gemcitabine of P-glycoprotein and multidrug resistance-associated protein-overexpressing human cancer cell lines. Br J Cancer 88: 1963–1970 12799644 10.1038/sj.bjc.6601011 PMC2741118

[bib5] Blay J, Gomez F, Sebban C, Bachelot T, Biron P, Guglielmi C, Hagenbeek A, Somers R, Chauvin F, Philip T (1998) The International Prognostic Index correlates to survival in patients with aggressive lymphoma in relapse: analysis of the PARMA trial. PARMA Group. Blood 92: 3562–3568 9808548

[bib6] Bredenfeld H, Franklin J, Nogova L, Josting A, Fries S, Mailander V, Oertel J, Diehl V, Engert A (2004) Severe pulmonary toxicity in patients with advanced-stage Hodgkin's disease treated with a modified bleomycin, doxorubicin, cyclophosphamide, vincristine, procarbazine, prednisone, and gemcitabine (BEACOPP) regimen is probably related to the combination of gemcitabine and bleomycin: a report of the German Hodgkin's Lymphoma Study Group. J Clin Oncol 22: 2424–2429 15136597 10.1200/JCO.2004.09.114

[bib7] Brice P, Bouabdallah R, Moreau P, Divine M, Andre M, Aoudjane M, Fleury J, Anglaret B, Baruchel A, Sensebe L, Colombat P (1997) Prognostic factors for survival after high-dose therapy and autologous stem cell transplantation for patients with relapsing Hodgkin's disease: analysis of 280 patients from the French registry. Societe Francaise de Greffe de Moelle. Bone Marrow Transplant 20: 21–26 9232251 10.1038/sj.bmt.1700838

[bib8] Burris III HA, Moore MJ, Andersen J, Green MR, Rothenberg ML, Modiano MR, Cripps MC, Portenoy RK, Storniolo AM, Tarassoff P, Nelson R, Dorr FA, Stephens CD, Von Hoff DD (1997) Improvements in survival and clinical benefit with gemcitabine as first-line therapy for patients with advanced pancreas cancer: a randomized trial. J Clin Oncol 15: 2403–2413 9196156 10.1200/JCO.1997.15.6.2403

[bib9] Canellos GP, Anderson JR, Propert KJ, Nissen N, Cooper MR, Henderson ES, Green MR, Gottlieb A, Peterson BA (1992) Chemotherapy of advanced Hodgkin's disease with MOPP, ABVD, or MOPP alternating with ABVD. N Engl J Med 327: 1478–1484 1383821 10.1056/NEJM199211193272102

[bib10] Canellos GP, Niedzwiecki D (2002) Long-term follow-up of Hodgkin's disease trial. N Engl J Med 346: 1417–1418 11986425 10.1056/NEJM200205023461821

[bib11] Chau I, Harries M, Cunningham D, Hill M, Ross PJ, Archer CD, Norman AR, Wotherspoon A, Koh DM, Gill K, Uzzell M, Prior Y, Catovsky D (2003) Gemcitabine, cisplatin and methylprednisolone chemotherapy (GEM-P) is an effective regimen in patients with poor prognostic primary progressive or multiply relapsed Hodgkin's and non-Hodgkin's lymphoma. Br J Haematol 120: 970–977 12648066 10.1046/j.1365-2141.2003.04226.x

[bib12] Chau I, Webb A, Cunningham D, Hill M, Rao S, Ageli S, Norman A, Gill K, Howard A, Catovsky D (2001) An oxaliplatin-based chemotherapy in patients with relapsed or refractory intermediate and high-grade non-Hodgkin's lymphoma. Br J Haematol 115: 786–792 11843810 10.1046/j.1365-2141.2001.03181.x

[bib13] Cheson BD, Horning SJ, Coiffier B, Shipp MA, Fisher RI, Connors JM, Lister TA, Vose J, Grillo-Lopez A, Hagenbeek A, Cabanillas F, Klippensten D, Hiddemann W, Castellino R, Harris NL, Armitage JO, Carter W, Hoppe R, Canellos GP (1999) Report of an international workshop to standardize response criteria for non-Hodgkin's lymphomas. NCI Sponsored International Working Group. J Clin Oncol 17: 1244 10561185 10.1200/JCO.1999.17.4.1244

[bib14] Coiffier B, Lepage E, Briere J, Herbrecht R, Tilly H, Bouabdallah R, Morel P, Van Den Neste E, Salles G, Gaulard P, Reyes F, Lederlin P, Gisselbrecht C (2002) CHOP chemotherapy plus rituximab compared with CHOP alone in elderly patients with diffuse large-B-cell lymphoma. N Engl J Med 346: 235–242 11807147 10.1056/NEJMoa011795

[bib15] Demidem A, Lam T, Alas S, Hariharan K, Hanna N, Bonavida B (1997) Chimeric anti-CD20 (IDEC-C2B8) monoclonal antibody sensitizes a B cell lymphoma cell line to cell killing by cytotoxic drugs. Cancer Biother Radiopharm 12: 177–186 10851464 10.1089/cbr.1997.12.177

[bib16] Dunsford ML, Mead GM, Bateman AC, Cook T, Tung K (1999) Severe pulmonary toxicity in patients treated with a combination of docetaxel and gemcitabine for metastatic transitional cell carcinoma. Ann Oncol 10: 943–947 10509156 10.1023/a:1008377819875

[bib17] Emmanouilides C, Colovos C, Pinter-Brown L, Hernandez L, Schiller G, Territo M, Rosen P (2004) Pilot study of fixed-infusion rate gemcitabine with Cisplatin and dexamethasone in patients with relapsed or refractory lymphoma. Clin Lymphoma 5: 45–49 15245607 10.3816/clm.2004.n.009

[bib18] Emmanouilides C, Jazirehi AR, Bonavida B (2002) Rituximab-mediated sensitization of B-non-Hodgkin's lymphoma (NHL) to cytotoxicity induced by paclitaxel, gemcitabine, and vinorelbine. Cancer Biother Radiopharm 17: 621–630 12537665 10.1089/108497802320970226

[bib19] Ferme C, Bastion Y, Lepage E, Berger F, Brice P, Morel P, Gabarre J, Nedellec G, Reman O, Cheron N, Oberlin O, Coiffer B (1995) The MINE regimen as intensive salvage chemotherapy for relapsed and refractory Hodgkin's disease. Ann Oncol 6: 543–549 8573532 10.1093/oxfordjournals.annonc.a059242

[bib20] Fisher RI, Gaynor ER, Dahlberg S, Oken MM, Grogan TM, Mize EM, Glick JH, Coltman Jr CA, Miller TP (1994) A phase III comparison of CHOP *vs*. m-BACOD *vs*. ProMACE-CytaBOM *vs*. MACOP-B in patients with intermediate- or high-grade non-Hodgkin's lymphoma: results of SWOG-8516 (Intergroup 0067), the National High-Priority Lymphoma Study. Ann Oncol 5(Suppl 2): 91–95 10.1093/annonc/5.suppl_2.s91 7515652

[bib21] Fossa A, Santoro A, Hiddemann W, Truemper L, Niederle N, Buksmaui S, Bonadonna G, Seeber S, Nowrousian MR (1999) Gemcitabine as a single agent in the treatment of relapsed or refractory aggressive non-Hodgkin's lymphoma. J Clin Oncol 17: 3786–3792 10577850 10.1200/JCO.1999.17.12.3786

[bib22] Friedberg JW, Neuberg D, Kim H, Miyata S, McCauley M, Fisher DC, Takvorian T, Canellos GP (2003) Gemcitabine added to doxorubicin, bleomycin, and vinblastine for the treatment of *de novo* Hodgkin disease: unacceptable acute pulmonary toxicity. Cancer 98: 978–982 12942565 10.1002/cncr.11582

[bib23] Germann N, Brienza S, Rotarski M, Emile JF, Di Palma M, Musset M, Reynes M, Soulie P, Cvitkovic E, Misset JL (1999) Preliminary results on the activity of oxaliplatin (L-OHP) in refractory/recurrent non-Hodgkin's lymphoma patients. Ann Oncol 10: 351–354 10355582 10.1023/a:1008310708853

[bib24] Grunewald R, Kantarjian H, Du M, Faucher K, Tarassoff P, Plunkett W (1992) Gemcitabine in leukemia: a phase I clinical, plasma, and cellular pharmacology study. J Clin Oncol 10: 406–413 1740680 10.1200/JCO.1992.10.3.406

[bib25] Guglielmi C, Gomez F, Philip T, Hagenbeek A, Martelli M, Sebban C, Milpied N, Bron D, Cahn JY, Somers R, Sonneveld P, Gisselbrecht C, Van Der Lelie H, Chauvin F (1998) Time to relapse has prognostic value in patients with aggressive lymphoma enrolled onto the Parma trial. J Clin Oncol 16: 3264–3269 9779700 10.1200/JCO.1998.16.10.3264

[bib26] Harris NL, Jaffe ES, Diebold J, Flandrin G, Muller-Hermelink HK, Vardiman J, Lister TA, Bloomfield CD (2000) The World Health Organization classification of hematological malignancies report of the Clinical Advisory Committee Meeting, Airlie House, Virginia, November 1997. Mod Pathol 13: 193–207 10697278 10.1038/modpathol.3880035

[bib27] Josting A, Rueffer U, Franklin J, Sieber M, Diehl V, Engert A (2000) Prognostic factors and treatment outcome in primary progressive Hodgkin lymphoma: a report from the German Hodgkin Lymphoma Study Group. Blood 96: 1280–1286 10942369

[bib28] Kewalramani T, Zelenetz AD, Nimer SD, Portlock C, Straus D, Noy A, O’Connor O, Filippa DA, Teruya-Feldstein J, Gencarelli A, Qin J, Waxman A, Yahalom J, Moskowitz CH (2004) Rituximab and ICE as second-line therapy before autologous stem cell transplantation for relapsed or primary refractory diffuse large B-cell lymphoma. Blood 103: 3684–3688 14739217 10.1182/blood-2003-11-3911

[bib29] Kouroussis C, Mavroudis D, Kakolyris S, Voloudaki A, Kalbakis K, Souglakos J, Agelaki S, Malas K, Bozionelou V, Georgoulias V (2004) High incidence of pulmonary toxicity of weekly docetaxel and gemcitabine in patients with non-small cell lung cancer: results of a dose-finding study. Lung Cancer 44: 363–368 15140550 10.1016/j.lungcan.2003.12.004

[bib30] Linch DC, Winfield D, Goldstone AH, Moir D, Hancock B, McMillan A, Chopra R, Milligan D, Hudson GV (1993) Dose intensification with autologous bone-marrow transplantation in relapsed and resistant Hodgkin's disease: results of a BNLI randomised trial. Lancet 341: 1051–1054 8096958 10.1016/0140-6736(93)92411-l

[bib31] Lister TA, Crowther D, Sutcliffe SB, Glatstein E, Canellos GP, Young RC, Rosenberg SA, Coltman CA, Tubiana M (1989) Report of a committee convened to discuss the evaluation and staging of patients with Hodgkin's disease: Cotswolds meeting. J Clin Oncol 7: 1630–1636 2809679 10.1200/JCO.1989.7.11.1630

[bib32] Manegold C, Bergman B, Chemaissani A, Dornoff W, Drings P, Kellokumpu-Lehtinen P, Liippo K, Mattson K, van Pawel J, Ricci S, Sederholm C, Stahel RA, Wagenius G, van Walree N, ten Bokkel-Huinink W (1997) Single-agent gemcitabine *versus* cisplatin-etoposide: early results of a randomised phase II study in locally advanced or metastatic non-small-cell lung cancer. Ann Oncol 8: 525–529 9261520 10.1023/a:1008207731111

[bib33] Marcus R, Imrie K, Belch A, Cunningham D, Flores E, Catalano J, Solal-Celigny P, Offner F, Walewski J, Raposo J, Jack A, Smith P (2003) CVP chemotherapy plus rituximab compared with CVP as first-line treatment for advanced follicular lymphoma. Blood 105: 1417–1423 10.1182/blood-2004-08-3175 15494430

[bib34] Moskowitz CH, Bertino JR, Glassman JR, Hedrick EE, Hunte S, Coady-Lyons N, Agus DB, Goy A, Jurcic J, Noy A, O’Brien J, Portlock CS, Straus DS, Childs B, Frank R, Yahalom J, Filippa D, Louie D, Nimer SD, Zelenetz AD (1999) Ifosfamide, carboplatin, and etoposide: a highly effective cytoreduction and peripheral-blood progenitor-cell mobilization regimen for transplant-eligible patients with non-Hodgkin's lymphoma. J Clin Oncol 17: 3776–3785 10577849 10.1200/JCO.1999.17.12.3776

[bib35] Noordijk E, Carde P, Hagenbeek A, Mandard A, Kluin-Nelemans JC (1997) Combination of radiotherapy and chemotherapy is advisable in all patients with clinical stage I–II Hodgkin's disease. Six-year results of the EORTC-GPMC controlled clinical trials H7-VF, H7-F and H7-U. Int J Radiat Oncol Biol Phys 39: 1739300752

[bib36] Peters GJ, Bergman AM, Ruiz van Haperen VW, Veerman G, Kuiper CM, Braakhuis BJ (1995) Interaction between cisplatin and gemcitabine *in vitro* and *in vivo*. Semin Oncol 22: 72–79 7481849

[bib37] Philip T, Armitage JO, Spitzer G, Chauvin F, Jagannath S, Cahn JY, Colombat P, Goldstone AH, Gorin NC, Flesh M, Laporte JP, Maraninchi D, Pico J, Bosly A, Anderson C, Schots R, Biron P, Cabanillas F, Dicke K (1987) High-dose therapy and autologous bone marrow transplantation after failure of conventional chemotherapy in adults with intermediate-grade or high-grade non-Hodgkin's lymphoma. N Engl J Med 316: 1493–1498 3295541 10.1056/NEJM198706113162401

[bib38] Philip T, Guglielmi C, Hagenbeek A, Somers R, Van der Lelie H, Bron D, Sonneveld P, Gisselbrecht C, Cahn JY, Harousseau JL, Coiffier B, Biron P, Mandelli F, Chauvin F (1995) Autologous bone marrow transplantation as compared with salvage chemotherapy in relapses of chemotherapy-sensitive non-Hodgkin's lymphoma. N Engl J Med 333: 1540–1545 7477169 10.1056/NEJM199512073332305

[bib39] Rose PG, Mossbruger K, Fusco N, Smrekar M, Eaton S, Rodriguez M (2003) Gemcitabine reverses cisplatin resistance: demonstration of activity in platinum- and multidrug-resistant ovarian and peritoneal carcinoma. Gynecol Oncol 88: 17–21 12504621 10.1006/gyno.2002.6850

[bib40] Roychowdhury DF, Cassidy CA, Peterson P, Arning M (2002) A report on serious pulmonary toxicity associated with gemcitabine-based therapy. Invest New Drugs 20: 311–315 12201493 10.1023/a:1016214032272

[bib41] Santoro A, Bredenfeld H, Devizzi L, Tesch H, Bonfante V, Viviani S, Fiedler F, Parra HS, Benoehr C, Pacini M, Bonadonna G, Diehl V (2000) Gemcitabine in the treatment of refractory Hodgkin's disease: results of a multicenter phase II study. J Clin Oncol 18: 2615–2619 10893294 10.1200/JCO.2000.18.13.2615

[bib42] Savage DG, Rule SA, Tighe M, Garrett TJ, Oster MW, Lee RT, Ruiz J, Heitjan D, Keohan ML, Flamm M, Johnson SA (2000) Gemcitabine for relapsed or resistant lymphoma. Ann Oncol 11: 595–597 10907954 10.1023/a:1008307528519

[bib43] Schmitz N, Pfistner B, Sextro M, Sieber M, Carella AM, Haenel M, Boissevain F, Zschaber R, Muller P, Kirchner H, Lohri A, Decker S, Koch B, Hasenclever D, Goldstone AH, Diehl V (2002) Aggressive conventional chemotherapy compared with high-dose chemotherapy with autologous haemopoietic stem-cell transplantation for relapsed chemosensitive Hodgkin's disease: a randomised trial. Lancet 359: 2065–2071 12086759 10.1016/S0140-6736(02)08938-9

[bib44] Sieber M, Franklin J, Tesch H (2002) Two cycles ABVD plus extended field radiotherapy is superior to radiotherapy alone in early stage Hodgkin's disease: Results of the German Hodgkin's Lymphoma Study Group (GHSH) trial HD7. Blood 100: 341 (abstract)12070046

[bib45] Therasse P, Arbuck SG, Eisenhauer EA, Wanders J, Kaplan RS, Rubinstein L, Verweij J, Van Glabbeke M, van Oosterom AT, Christian MC, Gwyther SG (2000) New guidelines to evaluate the response to treatment in solid tumors. European Organization for Research and Treatment of Cancer, National Cancer Institute of the United States, National Cancer Institute of Canada. J Natl Cancer Inst 92: 205–216 10655437 10.1093/jnci/92.3.205

[bib46] van Moorsel CJ, Pinedo HM, Veerman G, Bergman AM, Kuiper CM, Vermorken JB, van der Vijgh WJ, Peters GJ (1999) Mechanisms of synergism between cisplatin and gemcitabine in ovarian and non-small-cell lung cancer cell lines. Br J Cancer 80: 981–990 10362105 10.1038/sj.bjc.6690452 PMC2363050

[bib47] Velasquez WS, Cabanillas F, Salvador P, McLaughlin P, Fridrik M, Tucker S, Jagannath S, Hagemeister FB, Redman JR, Swan F, Barlogie B (1988) Effective salvage therapy for lymphoma with cisplatin in combination with high-dose Ara-C and dexamethasone (DHAP). Blood 71: 117–122 3334893

[bib48] Velasquez WS, McLaughlin P, Tucker S, Hagemeister FB, Swan F, Rodriguez MA, Romaguera J, Rubenstein E, Cabanillas F (1994) ESHAP – an effective chemotherapy regimen in refractory and relapsing lymphoma: a 4-year follow-up study. J Clin Oncol 12: 1169–1176 8201379 10.1200/JCO.1994.12.6.1169

[bib49] Zinzani PL, Bendandi M, Stefoni V, Albertini P, Gherlinzoni F, Tani M, Piccaluga PP, Tura S (2000) Value of gemcitabine treatment in heavily pretreated Hodgkin's disease patients. Haematologica 85: 926–929 10980630

